# Machine Learning Data Analysis Highlights the Role of *Parasutterella* and *Alloprevotella* in Autism Spectrum Disorders

**DOI:** 10.3390/biomedicines10082028

**Published:** 2022-08-19

**Authors:** Daniele Pietrucci, Adelaide Teofani, Marco Milanesi, Bruno Fosso, Lorenza Putignani, Francesco Messina, Graziano Pesole, Alessandro Desideri, Giovanni Chillemi

**Affiliations:** 1 Department for Innovation in Biological, Agro-Food and Forest Systems (DIBAF), University of Tuscia, 01100 Viterbo, Italy; 2 Institute of Biomembranes, Bioenergetics and Molecular Biotechnologies, IBIOM, CNR, 70126 Bari, Italy; 3 Department of Biology, University of Rome Tor Vergata, Via Montpellier 1, 00133 Rome, Italy; 4 Department of Biosciences, Biotechnology and Biopharmaceutics, University of Bari “A. Moro”, Piazza Umberto I, 1, 70121 Bari, Italy; 5 Unit of Microbiology and Diagnostic Immunology, Units of Microbiomics, Department of Diagnostic and Laboratory Medicine, Bambino Gesù Children’s Hospital, IRCCS, 00146 Rome, Italy; 6 Laboratory of Microbiology and Biological Bank National Institute for Infectious Diseases “Lazzaro Spallanzani” Istituto di Ricovero e Cura a Carattere Scientifico, 00149 Rome, Italy

**Keywords:** autism spectrum disorder, gut microbiota, dysbiosis, machine learning data analysis, *Parasutterella*, *Alloprevorella*, targeted metagenomics

## Abstract

In recent years, the involvement of the gut microbiota in disease and health has been investigated by sequencing the 16S gene from fecal samples. Dysbiotic gut microbiota was also observed in Autism Spectrum Disorder (ASD), a neurodevelopmental disorder characterized by gastrointestinal symptoms. However, despite the relevant number of studies, it is still difficult to identify a typical dysbiotic profile in ASD patients. The discrepancies among these studies are due to technical factors (i.e., experimental procedures) and external parameters (i.e., dietary habits). In this paper, we collected 959 samples from eight available projects (540 ASD and 419 Healthy Controls, HC) and reduced the observed bias among studies. Then, we applied a Machine Learning (ML) approach to create a predictor able to discriminate between ASD and HC. We tested and optimized three algorithms: Random Forest, Support Vector Machine and Gradient Boosting Machine. All three algorithms confirmed the importance of five different genera, including *Parasutterella* and *Alloprevotella*. Furthermore, our results show that ML algorithms could identify common taxonomic features by comparing datasets obtained from countries characterized by latent confounding variables.

## 1. Introduction

The role of the gut microbiota and its interaction with several organs’ physiology in pathological conditions has been extensively studied in recent years. The gut microbiota is involved in pathologies related to digestion, food intake, and energy metabolisms, such as Irritable Bowel Disease [1] and liver cirrhosis [2]. Through the interaction with the gut-brain axis, the microbiota is involved in several pathologies related to the central nervous system and the brain, such as Alzheimer’s and Parkinson’s disease [3]. The gut microbiota does not only affect the gut-brain axis in neurodegenerative diseases but also in neurodevelopmental conditions, such as the Autism Spectrum Disorders (ASD) [4]. Clinical observations suggest that the gut is involved in ASD physiology. In detail, ASD children are often characterized by gastrointestinal problems, such as constipation and diarrhea [5]. These symptoms are often present in gut dysbiosis conditions [6]. The influence of the gut microbiota in ASD has been extensively demonstrated in murine models [7]. For example, mice receiving a fecal transplant using the feces from ASD patients can develop an “autism-like” behavior [8] and a mouse model of autism shows a characteristic dysbiotic gut microbiota that may alter the tryptophan metabolism through the kynurenine pathway [9]. Since murine models provided this promising preliminary experimental evidence, a significant part of ASD literature focused on clinical implications for human patients. In fact, several studies have also been conducted on children with ASD, mainly sequencing the 16S marker gene from fecal samples to characterize the gut microbiota [10,11,12,13,14,15]. In all these studies, fecal samples from healthy controls (HC) were analyzed in order to highlight the dysbiosis between patients and controls and identify taxa possibly involved in the pathology. Dysbiosis could influence ASD pathology in several ways. For example, a reduction in metabolites synthesized by bacteria, such as Short Chain Fatty Acids (SCFAs), could be observed in ASD children [16,17]. Furthermore, gut dysbiosis has been correlated with the alteration of neurotransmitters, such as Gamma-aminobutyric acid (GABA) [17] and serotonin [12]. For instance, the lower abundance of GABA in ASD children was correlated with a higher abundance of the *Streptococcus* genus [17], while higher serotonin levels can be due to the lack of the *Bacteroides* genus [12]. Furthermore, a recent paper showed that the fecal concentrations of secretory IgA, zonulin and lysozyme were altered in ASD children, indicating a possible alteration of intestinal permeability [18].

Despite the significant evidence of the involvement of the gut microbiota in ASD, the literature presents discordant results. For example, the abundance of *Faecalibacterium* is significantly greater in ASD patients according to Ding and coauthors [19], while lower according to Coretti and coauthors [14]. Inconsistent results may arise from several factors, such as the different experimental and bioinformatic procedures used to analyze the data or sociodemographic aspects, such as the diet, that shape the gut microbiota [20]. Furthermore, in ASD children, peculiar eating behaviors, such as food selectivity and picky/fussy eating can be observed [21], leading to another confounder in the shape of the gut microbiota.

The need for a meta-analysis approach, capable of discriminating between perturbation of pathological origin and bias due to genetic, environmental (i.e., diet) or methodological nature is therefore clear. The first advantage of a meta-analytic work is the comparison of several studies conducted in particular methodological and environmental conditions. For example, a main aspect that could influence the single-study findings is the choice of controls. Depending on the controls’ characteristics (e.g., being inside or outside the patient’s family, being paired for gender or age), the reported differences between ASD patients and controls may vary. Apart from methodological issues, conducting a meta-analysis allows for the individuation of those bacteria that, despite the heterogeneity of the gut microbiota, the dominant effect of ambience in shaping it, and methodological choices, emerge as significant in several different studies. A synthetic, meta-analytic work highlighting such taxa is the first step toward individuating metabolic pathways that would explain the relationship between microbiota and the gut-brain axis within ASD patients and could be further experimentally explored.

This article describes a pooled analysis of eight publicly available datasets selected to identify bacterial genera as possible biomarkers for ASD pathology. All the datasets were analyzed using the same bioinformatic method, thus reducing one potential source of bias.

The application of Machine Learning (ML) algorithms has exploded in recent years for biomedical applications. These algorithms are useful for analyzing and integrating the large amount of data produced in the “omics” sciences [22], such as transcriptomics [23], metabolomics [24], and metagenomics [25]. The main advantages of ML algorithms are that they can identify subtle differences between classes, unlike classical statistics, and can also analyze very complex data [26]. ML has been applied to “omic” data related to ASD, mainly in metabolomics [27] and transcriptomics [28]. In the metagenomic field, a meta-analysis published in 2020 applied a Random Forest algorithm to ASD and identified three possible biomarkers (*Prevotella*, *Ruminococcus* and *Roseburia*) [29]. Our work differentiates from the previous, for three main reasons: (1) a higher number of classificators implemented in the analysis, to compare their performances and identify biomarkers with a higher degree of reliability; (2) a higher number of samples involved in the analysis, that allows for a better patient classification; (3) the use of the SHAP algorithm [30] to explain the importance of the bacteria involved in ASD.

To identify possible biomarkers for ASD, we used a ML approach on metagenomics 16S data, previously used to identify relevant taxa involved in different pathologies, such as Parkinson’s Disease [31] and Diabetes [32]. In particular, we used three algorithms: Random Forest (RF), Gradient Boosting Machine (GBM) and Support Vector Machine (SVM) [33]. In all three cases, the feature selection highlighted the importance of five genera. Of these, the *Parasutterella* and *Alloprevotella* genera caught our attention because they are strictly related to the *Sutterella* and *Prevotella* genera, which were previously identified as possible biomarkers of ASD [18,34].

## 2. Materials and Methods

The analysis strategy included bioinformatic data analysis, statistical data analysis, and machine learning data analysis as synthesized in Supplementary Figure S1 and detailed in the following paragraphs. 

### 2.1. Data Gathering and Bioinformatic Data Analysis

We selected seven datasets downloaded from public databases, which analyzed the gut microbiota in patients with ASD by sequencing the 16S marker gene. All the datasets were publicly available and involved controls. Seven datasets were associated with the following BioProject ID in the Sequence Read Archive database [35]: PRJNA355023 [10], PRJNA516054 [11], PRJNA453621 [12], PRJEB27306 [13], PRJEB29421 [14], PRJNA282013 [15], PRJNA754695 [18]. The dataset provided by the BioProject PRJNA516054 [11] contained both 16S and shotgun metagenomic data; only 16S samples were downloaded and analyzed. The raw sequence data were downloaded from the European Nucleotide Archive (www.ebi.ac.uk/ena (accessed on 1 November 2021). Furthermore, samples of 50 patients with ASD and 50 age and sex-matched controls from the American Gut Project [36] were downloaded using qiita (https://qiita.ucsd.edu/ (accessed on 1 November 2021). Additional samples for the Bioproject PRJNA754695 are related to the BBMRI (Biobanking and BioMolecular Resources Research infrastructure of Italy) biobank of the Bambino Gesù Pediatric Hospital. 

The number of samples and metadata information of all datasets are reported in Table 1 and Table S1. A Study Identifier (“Study ID”) was created for each dataset, using the first author’s name whenever possible. Regarding the American Gut Project samples, the “Study ID” chosen was “AGP”. All the calculations have been carried out at Cineca in the framework of the ELIXIR-IT HPC@CINECA program [37] and on the Tuscia-DIBAF HPC center.

The quality of sequencing data was assessed using FastQC [38]; the adapters were identified using fastp and eventually removed using cutadapt [39,40]. Bioinformatic data analysis was performed using the QIIME 2 pipeline [41]. Firstly, reads were quality filtered, chimera-checked and denoised in Amplicon Sequencing Variants (ASVs) using DADA2 [42]. Next, the taxonomy of representative sequences was assessed using the SILVA database [43] (version 132). Finally, representative sequences were aligned on the SILVA database using the blast+ tool provided by the QIIME 2 pipeline (classify-consensus-blast) [44], increasing the maximum hits to 50 (—p-maxaccepts parameter), to have a more precise taxonomic classification. Since all datasets used different 16S regions, the denoising and the taxonomic assignment procedures were performed independently for each dataset. The QIIME 2 Artifacts Data files (“.qza” data file extension) were generated for each dataset.

### 2.2. Statistical Software and Packages

Statistical and ML data analysis was performed using R vr. 3.5.3. The following R packages were used: phyloseq vr. 1.26., DESeq2 vr. 1.22.2, qiime2R vr. 0.99.12, ggplot2 vr. 3.2.1, plyr vr. 1.8.4, reshape2 vr. 1.4.3, scales 1.0.0, factoextra vr. 1.0.7, sva vr. 3.30.1, caret vr. 6.0.84, e1071 vr. 1.7.2, randomForest vr. 4.6.14, gbm vr. 2.1.8, shapper vr. 0.1.3, pROC vr. 1.15.3 and ROCR vr. 1.0.7 [30,45,46,47,48,49].

### 2.3. Data Filtering, Normalization, Multivariate Data Analysis and Batch-Effect Correction

The .qza files generated by the QIIME 2 pipeline were loaded in R 3.5.3. For each dataset, two .qza files were imported in R: the DADA2 ASVs table and the taxonomy table. In addition, a metadata file for each dataset was imported to link the sample name to the phenotype (ASD or HC). The datasets were imported using the qza_to_phyloseq function of the qiime2r package, generating a phyloseq object for each dataset. The phyloseq objects were merged using the merge_phyloseq function. 

Next, some filtering procedures were performed to remove low-quality ASVs and low-read count samples. Only samples with at least 1000 reads were analyzed. ASVs were filtered by abundance, removing ASVs with a relative abundance lower than 0.05% across all samples [50]. Data were normalized using DESeq2 [51] and collapsed to the genus level using the function tax_glom of the phyloseq package [45]. Finally, we applied a prevalence filter, only considering taxa present in at least the 10% of samples [52]. The filtering based on the number of reads was implemented using the prune_samples function; the one based on taxa prevalence at 10% was implemented using the filter_taxa function; both functions are available in the phyloseq package [45]. Finally, the filtering based on the abundance lower than 0.05% was implemented using anad hoc function in R.

Then, some multivariate data analyses were performed to evaluate how the gut microbiota was influenced by the status (ASD vs. HC) and whether the batch-effect emerged, since all studies were performed using different experimental procedures in different countries. The batch-effect was represented by the variable “Study ID”, which reflects the nationality, the environmental confounders that affect the gut microbiota (such as alimentation and social aspects), and the experimental procedures [53,54]. Methodological and environmental variables cannot be independently considered using the PERMANOVA test because of their collinearity. For this reason, we decided to use the Study ID to simultaneously consider both sources of background noise and take into account the batch effect across all samples.

Two different multivariate data analyses were performed: the Principal Coordinate Analysis (PCoA) and the Principal Component Analysis (PCA) [55]. The PCoA was used to estimate the β-diversity and perform statistical analysis, while the PCA was used to visualize the correlation among samples across all the datasets. Briefly, the PCoA analysis was performed by evaluating the Bray-Curtis dissimilarities using the distance function of the phyloseq package [45]. The first two principal coordinates were plotted using the ordinate and plot_ordination function of the phyloseq package [45]. The Bray-Curtis dissimilarity matrix [56] was used to evaluate the microbiota variance and its significance, explained by the Status (ASD or HC) and the Study ID. In order to obtain these values, a PERMANOVA test was performed using 9999 permutations using the adonis2 function [45]. Data were centered and scaled in order to perform the PCA using the prcomp function. 

The batch effect was reduced using the ComBat function of the SVApackage [47]. This function allows the estimation and removal of heterogeneity across the datasets. PCoA and PCA analyses were performed on data before and after applying the ComBat function. The variable used to adjust the batch effect was the “Study ID” as it represents the primary source of heterogeneity, as explained above. Both PCoA and PCA graphs were compared after the batch effect removal.

### 2.4. ML Algorithms: Optimization, Training and Evaluation

ML data analysis was performed on centered and scaled data, so that for each sample, each genus can take values from 0 to 1. Three different algorithms were chosen to create a microbiome-based classifier: random forest (“RF”), support vector machines (“SVM”) and gradient boosted machine (“GBM”). These algorithms were chosen on the basis of the literature on ML applied to the microbiota. The RF algorithm has been successfully applied for bacterial biomarker identification in fecal samples [57], while the SVM was able to classify the clinical profile of patients based on their microbiota [58]. Regarding the GBM, it was recently claimed to outperform other algorithms in this field of research [59].

The main goal of the classifiers is to identify the phenotype of a fecal sample (ASD or HC) by using the abundances of taxa identified as features. Independently by the applied algorithm, the procedure for algorithm training and optimization was divided into the following steps: (1) optimizing the algorithm parameters; (2) training and testing the algorithm using a k-fold procedure; (3) evaluating the algorithm performance; (4) identifying the optimal threshold, i.e., the threshold providing the same percentage of correct identification for ASD and HC samples and therefore two equal TPR and TNR values; this threshold was searched iteratively, computing at each iteration TPR and TNR.

The optimization of the algorithm parameters was performed using the expand.grid function and the train function of the caret package. This step allows the tuning of the hyper-parameters for each algorithm. The combination of the parameters which obtainthe best accuracy was selected to train and test the algorithm. The hyper-parameters to tune were: (1) the number of trees and the mtry parameter (number of variables to split each node) for the RF algorithm, (2) the C and sigma parameters for the SVM algorithm, (3) the number of trees, the minimal number of observations per node, the shrinkage parameters for the GBM algorithm. The lists of the parameter values tested for each algorithm are reported in Supplementary Table S2.

The “RF” algorithm was implemented using the function randomForest in the randomForest package, while the “GBM” algorithm was implemented using the gbm function in the gbm package. Regarding the “SVM” algorithm, it was implemented using the svm function in the e1701 package. 

The algorithms were trained and tested using a k-fold procedure. First, the dataset was divided into 5 folds (k = 5). Each fold consists of 80% of data used as the training set, whereas the remaining 20% is used as a test set. The k-fold cross-validation with a k-fold equal to 5 has been shown to maintain a low error rate, with low biases and low variance. Consequently, we performed a k-fold cross validation to evaluate the robustness of the method [60]. The training set consists of data in which the outcome label (ASD vs. HC) is given as input to the algorithm. Next, the algorithm is trained to recognize the outcome label using the features. Once the algorithm is trained, its performance is evaluated. The test set is a dataset of unseen data which are used to predict the outcome label. In the algorithm, the outcome label is considered “Positive” when corresponding to the ASD label and “Negative” when corresponding to the HC label. Finally, the predicted outcome label is compared to the real value. Briefly, after the test, a sample can be classified into four different classes:

- True Positive (TP): an ASD sample correctly predicted as an ASD sample;
- True Negative (TN): an HC sample correctly predicted as an HC sample;
- False Positive (FP): an ASD sample erroneously predicted as an HC sample;
- False Negative (FN): an HC sample erroneously predicted as an ASD sample.

The four classes are usually represented as a confusion matrix (Supplementary Table S3. Once the algorithm has been trained and tested, a confusion matrix is generated using the function confusionMatrix of the caret package [48]. The confusion matrix was used to compute the following metrics: accuracy, precision, True Positive Rate (Recall, Sensitivity), True Negative Rate (Specificity), and F-Score, calculated as follows [61]:

$$Accuracy=\frac{TP+TN}{TP+TN+FP+FN}$$

$$Precision=\frac{TP}{TP+FP}$$

$$True\;Positive\;Rate=Recall=Specificity=\frac{TP}{TP+FN}$$

$$True\;Negative\;Rate=Sensitivity=\frac{TN}{TN+FP}$$

$$F-score=\frac{2*Precision*Recall}{Precision+Recall}$$

Finally, we adjusted the probability of the classification to optimize the classifiers in recognizing the “Positive” class (the ASD samples) with the same probability of the “Negative” class (the HC samples). Usually, the classifier computes a probability that evaluates whether a sample can be predicted as a “Positive” sample. If the probability is higher than 50%, then the sample is predicted as a Positive class, otherwise it is predicted as a “Negative” class. Changing this probability threshold value can modify the True Positive and the True Negative Rate. We iteratively tested all the thresholds to identify the probability that led to TPR and TNR’s same value. This procedure allows building a model that can recognize ASD patients and controls with the same performance and represents the best compromise among all the tested models. The procedure to select the best probability threshold is reported in Supplementary Figure S2, which includes a graphical representation of the accuracy, TPR and TNR for each probability threshold. All metrics (TPR, TNR, Accuracy, Precision and F-score) were computed using all the samples, by summing up the values of TP, TN, FP and FN obtained by the confusion matrices created during the cross-validation.

### 2.5. Evaluation of Feature Importance, Feature Selection and Feature Contribution

The importance of each bacterial feature to the classification was evaluated during the algorithm training. Regarding the RF algorithms, the “Gini Impurity Decrease Index” was used to evaluate the importance of each feature to the classification [62]. In the SVM case, the weight matrix was computed to evaluate the feature importance [63]. The features were sorted from the most to the least important, and the algorithms were trained with the subset of the first n-th important features. In other words, the algorithm was re-trained systematically with a subset of the most important features to identify the lowest number of features that allows the classifier to perform as well as the one with all the features. This process is called “feature selection” and allows removing features that are not useful for the classification [31]. 

The feature importance is not the only relevant information that we can use to understand the role of each genus in the classification. The feature importance does not provide information on the contribution of each genus to the sample classification in ASD or HC samples. Actually, it only indicates whether the feature is important for the classification, but it does not reveal the contribution to the phenotype. In order to obtain this information, we applied the SHapley Additive exPlanations (SHAP) algorithm [30], which has been used to link the skin microbiota alteration to different phenotypes [64]. Briefly, the SHAP algorithm evaluates, for each sample, the contribution of a feature to the classification for a specific class. It allows calculating a parameter that can contribute to the class ASD or HC. In our study, values greater than 0 indicate that a feature contributes to classifying a sample as ASD. Otherwise, values below 0 indicate that the feature contributes to classifying a sample as HC. One of the main advantages of SHAP is the possibility to visualize the contribution to the classification of each taxon and its abundance simultaneously, allowing a direct graphical representation of the taxon’s role in the phenotype classification [64]. The function individual_variable_effect of the shapper package has been used to evaluate SHAP values. 

### 2.6. Dataset Analysis Based on Control Selection

After a preliminary data analysis on single datasets, we observed that datasets that selected controls outside the family context (neurotypical children) provide higher accuracy than the others (Section 3.3). Consequently, we repeated the data analysis by using three strategies, in order to evaluate the role of the control selection. In the first strategy, all the samples were used to train the classifier and evaluate the performance. In the second strategy, the dataset was reduced, including only the studies in which the controls were neurotypical children. In the second strategy, we only used the dataset provided by Vernocchi, AGP, Averina and Dan. The datasets by Son and Pulikkan were excluded from the analysis in the second strategy, since all the controls were siblings of the ASD patients [10,12]. In the third one, only the two datasets that used patient’s siblings as controls (i.e., Son and Pulikkan) were used to perform the machine learning classification.

## 3. Results

### 3.1. Number of Samples Analyzed and Preliminary Filters

We selected a total of 959 16S rRNA sequencing samples from public databases (see Section 2) in order to train ML classification models on individual phenotypes by using the bacterial abundances identified as a predictive feature. All the selected projects analyzed the fecal microbiota of children with ASD, compared to the microbiota of HC. Supplementary Table S1 reports detailed information on the aspects in each dataset that could act as confounders, such as experimental procedures and geographical locations of participants. The Illumina MiSeq sequencing technology was used in four datasets out of eight, while the Illumina HiSeq technology was used in two datasets (Averina and Dan) and one study used the Illumina NextSeq500 technology (Pulikkan). The majority of datasets used the QIAamp DNA Stool Mini Kit for DNA extraction, with the main exception of Zurita (FastDNA™ SPIN Kit for Soil), Son (ZR Fecal DNA MiniPre) and Son (#DP328, Tiangen Company, Beijing, China). The main experimental differences among the studies were related to the hypervariable sequence region, with a prevalence of the V3 and V4 regions, and a combination of them. The main exception was the Dan datasets, which include V1-V2 and V3-V4 reads.

**Figure 1:**
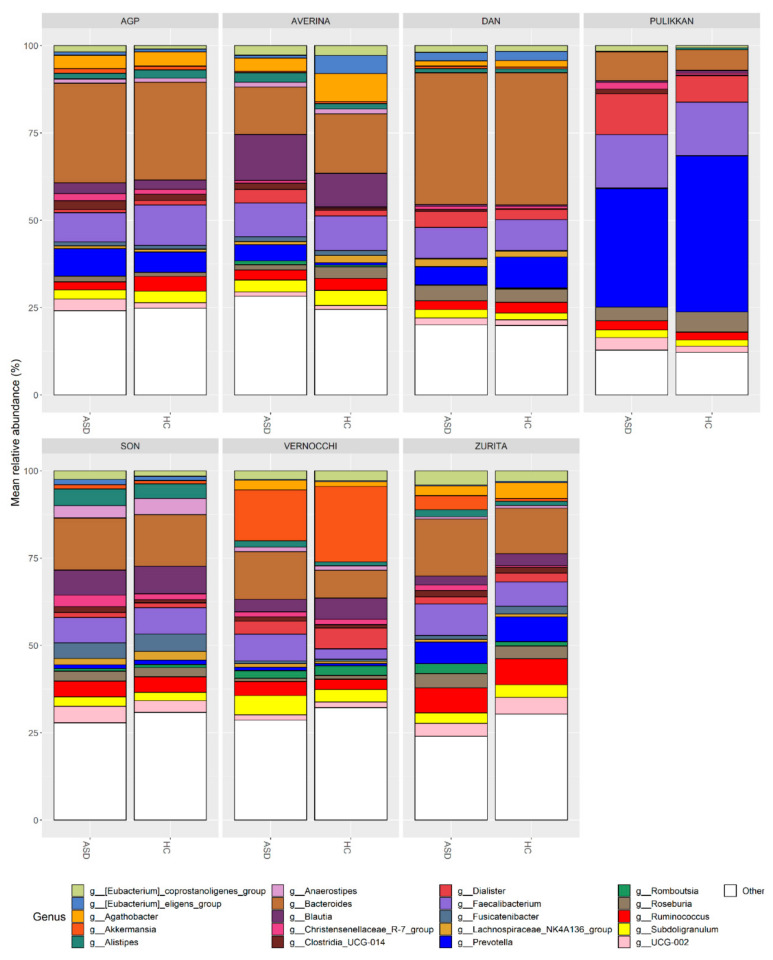
Relative abundance of the genus identified in all the datasets. For each dataset, the mean relative abundance of the 85 genera has been evaluated for ASD patients and HC. The 20 most abundant genera are represented using different colors and the remaining genera are reported in the “Others” bin.

We analyzed a total of 540 ASD and 419 HC samples using the QIIME2 pipeline (Table 1). First, we filtered out low abundance operational taxonomic units (ASVs) and samples with low read counts. After these filters, only four samples of the Coretti dataset were maintained. The number of samples pre- and post-filtering is represented in Supplementary Figure S3. Consequently, the Coretti dataset was removed from further analysis. Thus, the number of ASD samples was reduced to 521 and 402 for ASD and HC, respectively (Table 1). Overall, 85 genera were identified by QIIME2 on these samples and used as features to train and test three different ML algorithms. The relative abundance of the most representative genera is reported in Figure 1. Among the datasets, several differences related to genera abundances can be observed. For example, the Pulikkan dataset was characterized by a greater abundance of the *Prevotella* genus, with a greater abundance in HC samples. Instead, the Vernocchi dataset was enriched in the *Akkermansia* genus and the Dan, AGP, Son, Vernocchi and Zurita datasets showed a greater abundance of the *Bacteroides* genus. The *Blautia* genera were more represented in the Averina and Son datasets. The *Dialister* genus was more abundant in the Averina, Dan and Pulikkan datasets. The *Faecalibacterium* genus was more abundant in the AGP, Averina, Dan and Pulikkan datasets. Other abundant genera are *Agathobacter*, *Subdoligranulum* and *Roseburia*, identified with slight differences in all datasets. This graph highlighted high variability in the microbiota of ASD patients and controls among the datasets.

### 3.2. Analysis of Beta-Diversity and Evaluation of the Batch Effect

The structure of the microbial community (β-diversity) among ASD and HC samples was investigated by using a PERMANOVA test on a Bray-Curtis dissimilarity matrix computed by using all the samples. The β-diversity was also represented using a Principal Coordinate Analysis (PCoA). In addition, the correlations among samples were represented with a Principal Component Analysis (PCA). The results of the PERMANOVA test are reported in Table 2A. Two variables were analyzed: the phenotype (ASD vs. HC) and the Study ID. The Study ID is a variable that represents the methodological and environmental differences across samples. The main methodological differences are the sample management and storage, the DNA extraction kit and the 16S region analyzed (see Supplementary Table S1). Environmental variables reflect sociodemographic aspects, such as nationality and dietary habits. 

The pseudo-F, the R2 and the significance of the test are reported in Table 2A. Both variables (phenotype and Study ID) are statistically significant (*p* < 0.05), indicating that they shape the microbial community structure (Table 2A). However, the Study ID shows higher R2 values (0.29) than the phenotype (R2 = 0.03), indicating that it explains a larger portion of the variance in the dataset. Consequently, the methodological approaches and environmental variables can significantly affect the microbial community. The results of both PCA and PCoA reflect the differences in the bacterial genera among the dataset highlighted in Figure 1.

**Figure 2:**
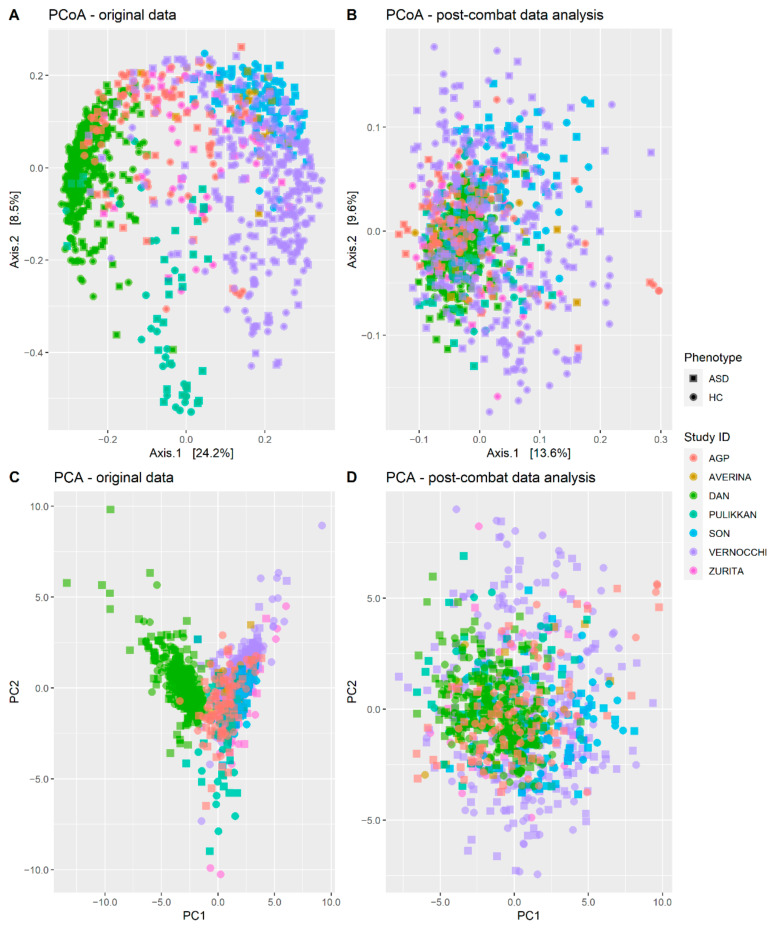
Principal Coordinate Analysis (PCoA) and Principal Component Analysis (PCA) were performed on microbial abundances. The phenotype variable is reported using a square or a circle for ASD and HC samples, respectively. Each color represents a different Project ID, namely one of the six datasets used in this study (AGP, Averina, Dan, Pulikkan, Vernocchi, Son and Zurita). (**A**) PCoA performed on original data; (**B**) PCoA performed after the removal of the batch effect using the ComBatfunction of the SVA package; (**C**) PCA performed on original data; (**D**) PCA performed after the removal of the batch effect using the ComBatfunction of the SVA package.

This concept is also represented by the PCoA (Figure 2A) and the PCA (Figure 2C), which show that samples obtained by the same experiment cluster together, independently from their phenotype. Nonetheless, the phenotype is significant using the PERMANOVA test. Consequently, there is a dysbiosis between ASD patients and HC, which is ‘masked’ by the alteration in microbial communities induced by the technical procedures that, in turn, can act as confounders.

Considering the role of the batch effect, we decided to use the ComBat function, from Surrogate Variable Analysis (SVA) R package, to reduce the batch effect among the datasets [47]. The removal of the batch effect is fundamental to reduce the differences among the datasets, thus creating a classifier able to distinguish ASD patients from HC. After removing the batch effect, we performed the PERMANOVA test on the Bray-Curtis dissimilarity matrix (Table 2B). As a result, the R2 related to the Study ID was reduced from 0.29 (Table 2A) to 0.14 (Table 2B). Instead, the R2 related to the “phenotype” variable was slightly reduced from 0.03 (Table 2A) to 0.025 (Table 2B). These results indicate that the batch effect was considerably reduced. Additionally, the PCoA (Figure 2B) and the PCA (Figure 2D) show a sparser clustering after removing the batch effect, indicating a reduction in the heterogeneity among the datasets. Consequently, we used the data transformed after removing the batch effect to perform the ML analysis.

### 3.3. Preliminary Results of the Random Forest on Three Datasets 

Three datasets with a sufficient number of samples were selected to perform some preliminary data analysis using the RF algorithm. We selected the Dan dataset, the Vernocchi dataset and the Son dataset. The main metrics of the algorithm performance are reported in Table 3. The Dan dataset provided the best metrics, with an accuracy and a precision greater than 0.80. In addition, the Recall (True Positive Rate) and the Specificity (True Negative Rate) were very high, with a value of 0.82. The Vernocchi dataset provided a precision comparable to the Dan dataset and an accuracy, Recall and Specificity values, equal to 0.72. On the other hand, the Son dataset yields the lower metrics, with an accuracy of 0.41, and a precision of 0.49. Furthermore, Recall and Specificity were both unsatisfactory (0.41). These different results could be partially attributed to different criteria for HC inclusion. In the Dan and Vernocchi datasets [12,18], the HC samples were selected among neurotypical children. On the contrary, the HC samples in the Son datasets were selected among patients’siblings. To further explore this factor, we decided to test the ML classifier by using three different strategies. In the first one, all the datasets were simultaneously analyzed. In the second one, we excluded the datasets in which HC were selected among patients’ siblings. Finally, in the third one, we performed the ML data analysis only using the two datasets that enrolled the HC controls among patients’ siblings.

### 3.4. Evaluation of Algorithm Metrics and Evaluation of the Control Selection Role

Three main algorithms were trained and tested using three sets of datasets: the first included all the datasets (Strategy 1), the second included only the datasets that did not admit patients’ siblings as controls (Strategy 2) and the third one included the datasets that admit patient’s siblings as controls (Strategy 3). In all three cases, the features (or predictors) used to train the algorithms were the 85 bacterial genera obtained with the QIIME2 pipeline in which the data were processed to remove the batch effect, as previously described, and standardized from 0 to 1. The phenotype variable (ASD or HC) was used as a target variable. In other words, a binary classifier was built using a supervised ML approach to predict if a fecal sample was related to an ASD or an HC patient.

The comparison among algorithms and strategies is reported in Table 4. The performances of the algorithms using only the datasets that did not admit patients’ siblings as controls were higher, compared to the other two analyses. In detail, the metrics that improved were the Recall (TPR)/Specificity (TNR), which means that the classifiers can better discriminate between patients and controls. Consequently, the metric that had the better improvement was the precision, since this strategy increased the number of TP and decreased the number of FP. Overall, the RF algorithm provided the best results. 

### 3.5. Feature Importance and Comparison of Features among Algorithms

We performed a feature selection to identify the minimal number of bacterial taxa with a predictive value close enough to the one obtained by using all the 85 genera. The underlying rationale is to remove features (i.e., genera) that can be considered “noisy” or “redundant” and that cannot contribute to ML model training. This process removes non-informative features (bacterial genera), i.e., features not used by the algorithm to perform the classification. The removal of uninformative features can highlight the importance of the bacterial genera involved in the pathology. Firstly, for each algorithm, all the features were sorted by importance by using the approach described in the Material and Methods section. Each feature was associated with a rank, which reflects its importance in the classification. The list of all ranks for each algorithm is reported in Supplementary Table S4. 

Subsequently, the algorithm was re-trained by using a subset of the first *n* most important features. Finally, we evaluated the precision metrics for all the subsets of trained algorithms. We decided to evaluate the precision since it was the metric with the greatest value when the algorithms were evaluated (Table 4). Furthermore, the precision considers the True Positives and the False Positives in its formula (Material and Methods section), allowing to consider simultaneously Recall (TPR) and Sensitivity (TNR). 

**Figure 3:**
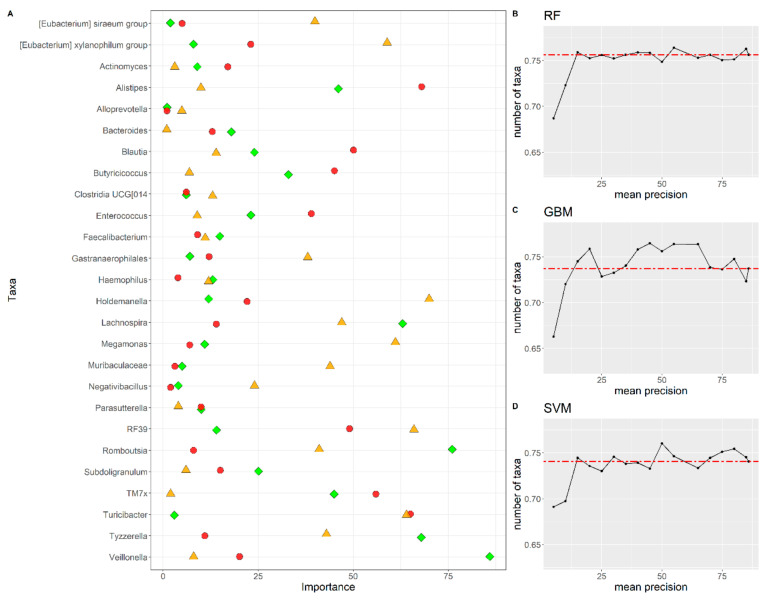
(**A**) Feature rank importance for all the 15 bacterial taxa identified by the feature selection procedure for the RF (green diamond), GBM (red circle) and SVM (orange triangle) algorithms. Feature selection for the (**B**) RF, (**C**) GBM and (**D**) SVM algorithms. On the y-axis, the mean precision value (evaluated on k = 5 fold) is reported. On the x-axis, the number of the n-th most relevant features used to train the algorithms is reported.

Algorithms trained with the 15 most important bacterial genera show a precision similar or identical to the ones trained with all the features and a cross-validation procedure with k = 5 (Figure 3B–D). The list of the 15 most important bacterial genera for each algorithm is reported in Table 5.

Using a Venn Diagram (Supplementary Figure S4), from the 15 most important bacterial genera for all the three algorithms, we identified five genera fundamental for the classification of all algorithms, i.e., *Alloprevotella*, *Sutterella*, *Haemophilus*, *Faecalibacterium* and an unclassified Clostridia “UCG 014”. When considering only the RF and the GBM algorithms, seven genera were important for the classification: *[Eubacterium]* siraeum_group, *Tyzzerella*, *Negativibacillus*, *Muribaculaceae*, *Gastranaerophilales*, *Megamonas* and *Rombustia*. In addition, the genus *Actinomyces* was identified as important by the RF and SVM algorithms. Finally, the *Bacteroides* and the *Subdoligranulum* were identified as important by the GBM and SVM algorithms. Some genera are identified only with specific algorithms. The genera *[Eubacterium]xylanophilum group* and *Holdemanella* were identified only by RF, while the genera *Lachnospira* were identified only by GBM. The SVM identified several genera not identified by other algorithms: *Agathobacter*, *Alistipes*, *Blautia*, *Butyricicoccus*, *Enterococcus*, TMx7 and *Veillonella*.

### 3.6. Feature Contribution to the ASD/HC Phenotype Classification

**Figure 4:**
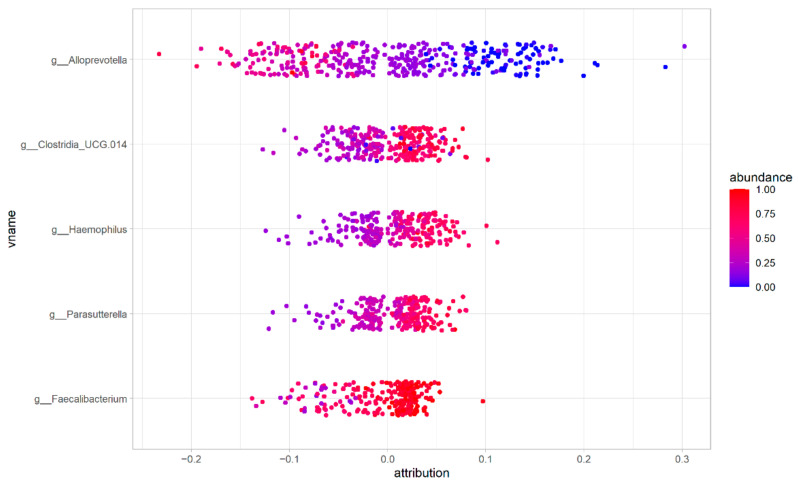
Results of the SHAP algorithm allow the visualization of the contribution of five features (bacterial genera) to classify a sample as ASD or HC. In this figure, each dot represents a sample, while the color indicates the microbial abundance. Red dots are samples for which a genus is abundant, while blue dots are genera that are poorly represented in a sample. Points that show an attribution greater than 0 are ASD samples, while points that show an attribution lower than 0 are HC samples.

Using the SHAP algorithm, we evaluated the role of the bacterial genera in the classification. Figure 4 shows how the five bacterial genera identified by all the algorithms contribute to the ASD or HC. In each row, a genus is represented. Each dot represents a sample, and its color reflects its abundance. If a sample shows an attribution greater than 0, it is an ASD sample, otherwise is an HC sample.

For example, lower abundances of *Alloprevotella* genus contribute to the classification as an ASD sample (abundance in ASD samples = 0.34 ± 0.20, abundance in HC samples = 0.12 ± 0.14). Conversely, greater abundances of *Parasutterella* (ASD samples = 0.57 ± 0.16, HC samples = 0.38 ± 0.17), *Haemophilus* (ASD samples = 0.57 ± 0.19, HC samples = 0.33 ± 0.17), *Faecalibacterium* (ASD samples = 0.86 ± 0.14, HC samples = 0.70 ± 0.21) and an unclassified Clostridiales UCG 14 (ASD samples = 0.60 ± 0.21, HC samples = 0.34 ± 0.17) contribute to the classification as an ASD sample. 

## 4. Discussion

In this study, we present a pooled bioinformatic data analysis on eight publicly available 16S datasets related to the fecal microbiota of ASD patients [10,11,12,13,14,15,18]. This work’s main goal is to harmonize the bioinformatic data analysis of the data produced in different laboratories, minimizing the background noise due to environmental and methodological variables, and creating an ML classifier that can help the identification of potential prokaryotes involved in the pathology. The fastq files from the SRA Bioproject linked to these studies represent a dataset with 959 samples (540 ASD and 419 HC).

Firstly, we filtered low abundant taxa and evaluated the relative abundances of the main genera identified in ASD and HC samples in all datasets (Figure 1). This representation showed several differences among datasets in the relative abundance of different taxa. For example, the *Prevotella* genus showed a greater abundance in HC samples, in line with the result reported by Pulikkan et al. [10]. Instead, the *Dialister* genus was more abundant in the Dan dataset, with a greater abundance in the ASD group, as reported by Dan et al. [12]. Not all the results from the previous studies were reproduced by our analysis. For example, the increased abundance in *Bacteroidetes* composition in ASD was reported by Dan et al. [12]. These discrepancies can be due to the different bioinformatic approaches used to identify the taxa, such as the reference database used for taxonomic classification. These analyses highlighted several differences in the microbiota composition in all the datasets.

Subsequently, we performed a multivariate analysis using the PCoA and the PCA. Both techniques showed that samples were clustered by individual studies (Figure 2A,C). This result is in line with a recent pooled analysis performed on 16S data on diabetic patients and healthy individuals [32] and reflected the differences in the relative abundance of taxa among datasets (Figure 1). As observed by Que et al., this clustering can be due to different aspects, such as the different experimental procedures and sociodemographic and behavioral factors (ethnicity and diet habits) [32]. Consequently, we transformed the data to remove the batch effect (Figure 2B,D) and performed a classification using three different ML algorithms. 

Our analysis highlighted the importance of the control choice. We obtained better classification performances, in fact, by using a subset of studies in which the HC were selected outside the family context (Table 3). This result is in line with a recent study, which investigated the microbiota using a proteomic approach in fecal samples from ASD patients, healthy relatives (siblings) and unrelated HC [65]. The taxa distribution was more variable between ASD and unrelated HC, while it was less variable between ASD and healthy relatives [65]. Furthermore, it has been shown that the beta-diversity between cohabiting twins is lower compared to the beta-diversity between not cohabiting twins [66]. The cohabitation and shared dietary habits between ASD children and their siblings can shape the gut microbiota, making it more similar and less variable. Consequently, the ML classifiers are not able to distinguish between the ASD and HC classes in this case.

After completing a feature selection, all the algorithms identified five genera important for a correct classification of the sample as ASD or HC. We mainly analyzed *Alloprevotella* and *Parasutterella* since they are strictly related to *Prevotella* and *Sutterella*. Both *Prevotella* and *Sutterella* were shown to be altered in ASD disorder [17,67]. It is worth noting that identifying these two strictly related genera by the ML analysis could be of scientific and clinical interest, directing the research towards the study of less known bacterial species of the human microbiota. Moreover, this meta-analysis also identified other bacteria such as *Heamophilus*, *Faecalibacterium* and Clostridia that were previously found altered in ASD children [17,19,68]. Thus, the three classifiers individuated not only new interesting taxa, but even genera that are already known in ASD gut dysbiosis. Furthermore, other bacteria previously reported were identified as highly important by a single or two classifiers, such as *Bacteroides* [12], *Actinomyces* [69], *Eubacterium* [70], *Subdoligranulum* [71] and *Veillonella* [72].

Our data showed that the lower levels of the *Alloprevotella* genus contribute to classifying the sample as an ASD (Figure 4). This is in line with a recent secondary data analysis on the shotgun dataset of Averina et al. [11], which showed that the *Alloprevotella* genus is completely absent in ASD patients [73]. The *Alloprevotella* genus is considered a beneficial bacteria, able to produce butyric acid [74],which is reduced in ASD patients [70]. The *Alloprevotella* genus may promote an anti-inflammatory environment [75]. Thus, its reduction in ASD children may increase gut inflammation. The family *Prevotellaceae* is reduced in ASD patients [4] and includes four genera (*Prevotella*, *Alloprevotella*, *Halella* and *Paraprevotella*) [76]. The reduction in the *Prevotella* genus contributes to classifying the sample as an ASD, with a trend similar to the *Alloprevotella* genus. The lower abundance of *Prevotella* in ASD children has been confirmed in other studies [17,18,67]. The different abundance of *Prevotella*, as well as *Firmicutes* and *Clostridiales,* were observed in ASD patients as compared to HC [77]. Notably, lower levels of the *Prevotella* genus have been correlated with vitamin B1 deficiency [78]. On the other hand, the abundance of *Prevotella* has been associated with colorectal carcinoma [79]and ileal Crohn’s disease [80], inducing changes in the expression profile of colon-rectal cells [81].

The *Parasutterella* genus is strictly related to the *Sutterella* genus [82]. Our data show that higher abundances contribute to classifying a sample as an ASD (Figure 4). Similar results were confirmed in other studies, in which the *Parasutterella* genus has been found with higher abundances in ASD patients [12,17,71], as well as in patients with the depressive disorder [83,84]. The *Parasutterella* genus is correlated to gut functionality. In detail, higher levels of *Parasutterella* are related to intestinal and chronic inflammation in IBS [85]. Notably, the levels of *Parasutterella* were higher in ASD patients with abdominal pain [86]. *Parasutterella* abundance is inversely correlated to metabolic processes linked to a high-fat diet [87,88].

Thus, the potential correlation between these taxa and ASD would suggest their engagement with metabolic pathways, which could modulate the microbiota-gut-brain axis.In fact, tryptophan is an essential amino acid, the precursor of the neurotransmitter serotonin, which plays an essential role in psychiatric disorders [89], and their metabolism could be influenced by gut microbial composition [90]. 

In this context, *Parasutterella*, *Alloprevotella* and *Prevotella* genera are an active part in these biological processes [83,84]. *Parasutterella* genus abundance was associated with fecal metabolic profile modification, such as tryptophan, tyrosine, bilirubin, purine, and bile acid metabolism [91]. In *Prevotella* genus, the metabolizing activity of tryptophan into indole was observed [92]. *Prevotella* abundance, when compared to *Bacteroides* level (P/B ratio), was inversely related to fecal tryptophan [93]. The related genus *Alloprevotella* has been shown to be involved in the tryptophan metabolism in murine models, with a negative correlation with tryptophan [94] and a positive correlation with tryptophan metabolites [95]. 

In conclusion, the ML-based bioinformatic pipeline, applied to 16S datasets related to the fecal microbiota of ASD patients, allowed to remove the background noise and unravel the possible signature of microbiome related to ASD. The main taxa used by the classifiers to discriminate between ASD and HC are *Alloprevotella* and *Parasutterella*, both reduced in ASD patients and correlated with inflammation and tryptophan metabolism. Further analyses are needed to elucidate the role of these two genera in ASD patients. It is unclear if the dysbiosis of the microbiota in ASD patients can be a consequence or a cause for the pathology, and the cause-effect mechanisms remain unclear [96]. In both cases, their value as predictor of the ASD pathology is high. Nevertheless, the correlation of these genera with ASD, identified with ML machine models, can be considered the first step to further elucidating the biology of these bacteria in this complex disease. The availability of more datasets, relating microbiome with clinical profiles, could allow further refining of the ML models, allowing to profile ASD patients, not limiting them to the ASD vs. HC classification. This would be a step forward toward microbiome-based personalized medicine [97] regarding the treatment of patients with ASD [98].

Our reanalysis again demonstrates, if needed, the importance of sharing clinical data with public databases. As previously observed, too many clinical articles are still published without the relative sample codes or are deposited with incorrect labels [99,100] and private requests remain unanswered. This is not only a limit to the reproducibility of published results but, as demonstrated by this study and several other meta-analyses, new important biological knowledge can be produced with ML and AI (Artificial Intelligence) approaches. However, larger datasets that can take into account socio-economical, environmental and host genetic factors are needed to create more accurate ML predictors.

## Figures and Tables

**Table 1:** Dataset used in this study. For each study, the number of ASD samples and HC samples before and after the filtering are reported. In addition, whenever possible, the BioProject ID is reported. For each study, a Study ID was defined, using the first Author Name or AGP for samples downloaded by the American Gut Project [36].

Study ID	ASD Samples	HC Samples	ASD Samples after Quality Filtering	HC Samples after Quality Filtering	Country	BioProject ID
Averina	15	5	15	5	Russia	PRJNA516054
Coretti	11	14	4	0	Italy	PRJEB29421
Dan	142	143	142	143	China	PRJNA453621
Pulikkan	30	24	30	24	India	PRJNA355023
Son	59	44	59	44	USA	PRJNA282013
Zurita	27	31	27	31	Ecuador	PRJEB27306
Vernocchi	206	108	197	108	Italy	PRJNA754695
AGP	50	50	47	47	USA	-

**Table 2:** Pseudo-Fscore (F), Degree of Freedom (Df), Sum of Variance, R2 and *p*-value of PERMANOVA test conducted on microbial communities. Two variables were analyzed: the phenotype (ASD vs. HC) and the Study ID, which represent an identifier for each dataset (AGP, Averina, Dan, Pulikkan, Vernocchi, Son, Zurita). In (**A**) the statistics prior to the batch effect removal using combat are reported. In (**B**) the values after the removal of batch effect are reported.

Variable	Df	Sum	R2	F	Pr (>F)
(A) Values prior to batch effect removal					
Phenotype	1	0.0788	0.03050	39.354	0.001
ìStudy ID	6	74.081	0.28705	616.687	0.001
Residual	911	182.396	0.70676		
Total	918	328.85	1.0000		
**(B) Values after to batch effect removal**					
Phenotype	1	0.0782	0.02508	27.416	0.001
Study ID	6	42.040	0.13874	255.534	0.001
Residual	911	259.968	0.85795		
Total	918	303.012	1.000		

**Table 3:** Comparison of the RF performance on three datasets. The algorithm parameters for each dataset were selected by using a grid search approach and the values which provided the greatest accuracy were selected. The threshold indicates the probability value at which the Recall (True Positive Rate, TPR) and the Specificity (True Negative Rate, TNR) were the same (see Section 2 and Supplementary Figure S2 for more details on this procedure). The following metrics are reported: Accuracy, Precision, Recall (TPR), Specificity (TNR) and F-score.

Dataset	Algorithm Parameters	Threshold	Accuracy	Precision	Recall (TPR) & Specificity (TNR)	F-Score
**Dan** [12]	ntree = 1500,	0.4540	0.85	0.85	0.86	0.85
	mtry = 10.21					
**Vernocchi** [18]	ntree = 1000,	0.6190	0.72	0.82	0.72	0.77
	mtry = 10.21					
**Son** [15]	ntree = 1500,	0.5640	0.41	0.49	0.41	0.44
	mtry = 6.21					

**Table 4:** Comparison of the algorithm performance using three strategies. In Strategy 1, all the datasets were used to train and test the algorithms. In Strategy 2,the dataset that did not admit patients’ siblings were used to train and test the algorithms. In Strategy 3, the two datasets that admit the patients’ siblings were used to train and test the algorithm. The threshold indicates that the probability value at which the Recall (True Positive Rate, TPR) and the Specificity (True Negative Rate, TNR) were the same (see Section 2 and Supplementary Figure S2 for more details on this procedure). The following metrics are reported: Accuracy, Precision, Recall (TPR), Specificity (TNR) and F-score.

Algorithm	Strategy	Algorithm Parameters	Threshold	Accuracy	Precision	Recall (TPR) & Specificity (TNR)	F-Score
RF	1	ntree = 500,	0.5580	0.67	0.71	0.67	0.70
		mtry = 7.21					
RF	2	ntree = 2000,	0.5570	0.70	0.76	0.70	0.72
		mtry = 12.27					
RF	3	ntree = 500,	0.5640	0.49	0.54	0.54	0.54
		mtry = 8.21					
GBM	1	n.trees = 1000,	0.6545	0.62	0.68	0.62	0.65
		interaction.depth = 1,					
		n.minobsinnod = 1,					
		shrinkage = 0.1					
GBM	2	n.trees = 1000,	0.6053	0.69	0.73	0.69	0.71
		interaction.depth = 1,					
		n.minobsinnod = 5,					
		shrinkage = 0.1					
GBM	3	n.trees = 2500,	0.9853	0.48	0.54	0.49	0.47
		interaction.depth = 1,					
		n.minobsinnod = 0.1,					
		shrinkage = 20					
SVM	1	C = 1, sigma = 2.9802 × 10^−8^	0.5966	0.65	0.70	0.65	0.67
SVM	2	C = 246, sigma = 3.1250 × 10^−2^	0.6025	0.69	0.74	0.70	0.72
SVM	3	C = 81, sigma = 9.7656 × 10^−4^	0.5632	0.45	0.53	0.49	0.50

**Table 5:** Feature importance for the RF, GBM and SVM algorithms. For each algorithm, the rank of the 15 most important bacterial genera is reported. The 15 most important bacterial genera were identified by a feature selection procedure. Blank spaces indicate that specific genera were not identified among the 15th most important in the feature selection for a specific algorithm.

Bacterial *Taxa*	Importance “RF” Algorithm	Importance “GBM” Algorithm	Importance “SVM” Algorithm
*Alloprevotella*	1	1	5
*Clostridia UCG-014*	6	6	13
*Faecalibacterium*	15	9	11
*Haemophilus*	13	4	12
*Parasutterella*	10	10	4
*[Eubacterium] siraeum group*	8	5	
*Actinomyces*	9		3
*Bacteroides*		13	1
*Gastranaerophilales*	7	12	
*Megamonas*	11	7	
*Muribaculaceae*	5	3	
*Negativibacillus*	4	2	
*Subdoligranulum*		15	6
*[Eubacterium] xylanophilum group*	2		
*Agathobacter*			15
*Alistipes*			10
*Blautia*			14
*Butyricicoccus*			7
*Enterococcus*			9
*Holdemanella*	12		
*Lachnospira*		14	
*RF39*			
*Romboutsia*	14	8	
*TM7x*			2
*Turicibacter*			
*Tyzzerella*	3	11	
*Veillonella*			8

## Data Availability

Not applicable.

## Supplementary Materials

The following supporting information can be downloaded at: https://www.mdpi.com/article/10.3390/biomedicines10082028/s1, Supplementary Table S1. Detailed information of the dataset used in this study downloaded from the SRA database; Supplementary Table S2. List of parameters and values evaluated using a Grid Search procedure; Table S3. Confusion matrix used to evaluate algorithm performance; Supplementary Table S4. Feature importance for the Random Forest (RF), Gradient Boosting Machine (GBM) and Support Vector Machines (SVM). For each algorithm, the importance of each bacterial genera (feature) was evaluated. The features were sorted by using a rank, which reflects the importance of the taxa for each algorithm. For example, the feature with rank 1 is the most important for the algorithm, then the second most important has a rank equal to 2; Supplementary Figure S1. Flowchart of the analysis strategy implemented in the study; Supplementary Figure S2. Graphical representation of the procedure used to select the best probability threshold (cutoff) for the classifiers; Supplementary Figure S3. Number of samples for each dataset pre and post filtering procedure; Supplementary Figure S4. Venn Diagram of the feature identified by each algorithm.

## Abbreviations

AGP	American Gut Project
ASD	Autism Spectrum Disorder
ASVs	Amplicon Sequence Variants
FN	False Negative
FP	False Positive
GABA	Gamma-aminobutyric acid
GBM	Gradient Boosting Machine
HC	Healthy Controls
ML	Machine Learning
PCA	Principal Coordinate Analysis
PCoA	Principal Component Analysis
RF	Random Forest
SCFAs	Short Chain Fatty Acids
SHAP	SHapley Additive exPlanations algorithm
SVA	Surrogate Variable Analysis
SVM	Support Vector Machine
TN	True Negative
TNR	True Negative Rate
TP	True Positive
TPR	True Positive Rate
