# Supplementary material for: Machine Learning Data Analysis Highlights the Role of Parasutterella and Alloprevotella in Autism Spectrum Disorders
Source: Biomedicines. 2022 Aug 19;10(8):2028. doi: 10.3390/biomedicines10082028 (PMC9405825; doi:10.3390/biomedicines10082028)
Supplement: Supplementary file 1 [file biomedicines-10-02028-s001.zip › biomedicines-1802766-supplementary.pdf]

Supplementary Tables

Supplementary Table S1. Detailed information of the dataset used in this study downloaded from the SRA database.

BioProject	Reference	Year	Country	ASD patients	Healthy controls	Age (years)	Control selection	DNA extraction kit	Sequencing	16S Region	Database
**PRJEB27306**	[13]	2019	Ecuador	25	35	5-12	Neurotypical children (same school in the same metropolitan district)	FastDNA™ SPIN Kit for Soil (MP Biomedicals, USA)	Illumina MiSeq	V4	GreenGenes
**PRJEB29421**	[14]	2018	Italy	11	14	2-4	Neurotypical children (visited the center for minor surgical operations)	QIAamp DNA Stool Mini Kit (Qiagen, CA, USA)	Illumina MiSeq	V3-V4	GreenGenes
**PRJNA282013**	[15]	2015	USA	59	44	7-14	Neurotypicalsibilings	ZR Fecal DNA MiniPre(Zymo Research Corporation, Irvine, CA)	Illumina MiSeq	V1-V2 V2-V3	Silva 115NR99
**PRJNA355023**	[10]	2018	India	30	24	3-16	Neurotypical children of the same family	QIAampStool Mini Kit (Qiagen, CA, USA)	NextSeq500	V3	GreenGenes
**PRJNA453621**	[12]	2020	Cina	143	142	2-13	Neurotypicalchildren (from kindergartens)	DNA extraction kit (#DP328, Tiangen Company, Beijing, China)	Illumina HiSeq	V4	GreenGenes
**PRJNA754695**	[18]	2022	Italy	206	108	3-15	Neurotypical children	QIAampStool Mini Kit (Qiagen, CA, USA)	Illumina MiSeq	V3-V4	GreenGenes
**PRJNA516054**	[11]	2020	Russia	15	5	3-5	Neurotypical children	QIAampStool Mini Kit (Qiagen, CA, USA)	Illumina HiSeq	V3-V4	-

Supplementary Table S2. List of parameters and values evaluated using a Grid Search procedure

Algorithm	*Parameter*	*Number of ASD samples*
Random Forest	*Number of trees*	*500, 1000, 1500, 2000, 2500*
	Mtry	*from sqrt(number taxa)-3 to sqrt(number taxa)+3*
Support Vector Machine	*C*	*1, 2, 3, 4, 8, 9, 16, 27, 32, 81, 243*
	Sigma	*2^-25, 2^-20, 2^-15, 2^-10, 2^-5, 2^0*
Gradient Boosting Machine	*Number of trees*	*500, 1000, 1500, 2000, 2500*
	Minimal number of observation per node	*1, 5, 10, 15, 20*
	Shrinkage	*0.001, 0.0001, 0.01, 0.1*
	Interaction depth	*1*

Supplementary Table S3. Confusion matrix used to evaluate algorithm performance.

Predicted as	***HC (0)***	***ASD (1)***
HC (0)	***True Negative***<br>*An HC sample correctly predicted as an HC sample*	***False Positive***<br>*An HC sample erroneously predicted as an ASD sample*
ASD (1)	***False Negative***<br>*An ASD sample erroneously predicted as an HC sample*	***True Positive***<br>*An ASD sample correctly predicted as an ASD sample*

Supplementary Table S4. Feature importance for the Random Forest (RF), Gradient Boosting Machine (GBM) and Support Vector Machines (SVM). For each algorithm, the importance of each bacterial genera (feature) was evaluated. The features were sorted by using a rank, which reflects the importance of the taxa for each algorithm. For example, the feature with rank 1 is the most important for the algorithm, then the second most important have a rank equal to 2.

**Bacterial taxa**	**Importance "RF" algorithm**	**Importance "GBM" algorithm**	**Importance "SVM" algorithm**
Alloprevotella	1	1	5
[Eubacterium] siraeum group	2	5	40
Turicibacter	3	65	64
Negativibacillus	4	2	24
Muribaculaceae	5	3	44
ClostridiaUCG[014	6	6	13
Gastranaerophilales	7	12	38
[Eubacterium] xylanophilum group	8	23	59
Actinomyces	9	17	3
Parasutterella	10	10	4
Megamonas	11	7	61

Holdemanella	12	22	70
Haemophilus	13	4	12
RF39	14	49	66
Faecalibacterium	15	9	11
Romboutsia	76	8	41
Tyzzerella	68	11	43
Bacteroides	18	13	1
Lachnospira	63	14	47
Subdoligranulum	25	15	6
Anaerostipes	21	16	52
Lachnospiraceae UCG-004	38	18	16
Enterorhabdus	37	19	20
Veillonella	86	20	8
NK4A214 group	27	21	42
Neisseria	69	24	86
Dialister	26	25	29
UCG-002	79	26	49
[Ruminococcus] gauvreauii group	48	27	23
Lachnospiraceae NK4A136 group	42	28	67
Collinsella	31	29	78
[Clostridium] innocuum group	20	30	18
TM7x	45	56	2
Butyricicoccus	33	45	7
Enterococcus	23	39	9
Alistipes	46	68	10
Blautia	24	50	14
Agathobacter	51	54	15
Streptococcus	32	80	17
Corynebacterium	53	66	19
Clostridia vadinBB60 group	41	40	21
Lachnospiraceae UCG-001	30	61	22
Sutterella	60	79	25
Parvimonas	75	76	26
[Ruminococcus] gnavus group	66	74	27
Porphyromonas	34	59	28
Gemella	82	86	30
Prevotella	57	64	31
Ruminococcus	83	72	32
Bifidobacterium	43	63	33
Lachnoclostridium	71	57	34

Peptoniphilus	47	47	35
Monoglobus	40	67	36
Fusicatenibacter	65	71	37
Phascolarctobacterium	85	58	39
Roseburia	77	53	45
UCG-003	35	77	46
IncertaeSedis	49	69	48
[Eubacterium] eligens group	52	37	50
Lactobacillus	17	83	51
Colidextribacter	61	70	53
[Eubacterium] ruminantium group	56	32	54
Granulicatella	78	78	55
GCA-900066575	70	85	56
Erysipelatoclostridium	39	41	57
Barnesiella	55	46	58
Oscillibacter	62	62	60
Clostridium sensustricto 1	36	34	62
Fusobacterium	80	55	63
[Ruminococcus] torques group	59	42	65
UCG-005	16	31	68
Terrisporobacter	22	82	69
[Eubacterium] hallii group	72	52	71
Prevotellaceae NK3B31 group	84	48	72
Parabacteroides	64	33	73
Christensenellaceae R-7 group	28	43	74
Akkermansia	44	73	75
Fenollaria	54	38	76
Coprococcus	50	84	77
UCG-010	19	36	79
[Eubacterium] coprostanoligenes group	58	44	80
Erysipelotrichaceae UCG-003	73	75	81
Dorea	67	60	82
Family XIII AD3011 group	81	81	83
[Eubacterium] ventriosum group	74	51	84
Lachnospiraceae ND3007 group	29	35	85

Supplementary Figures

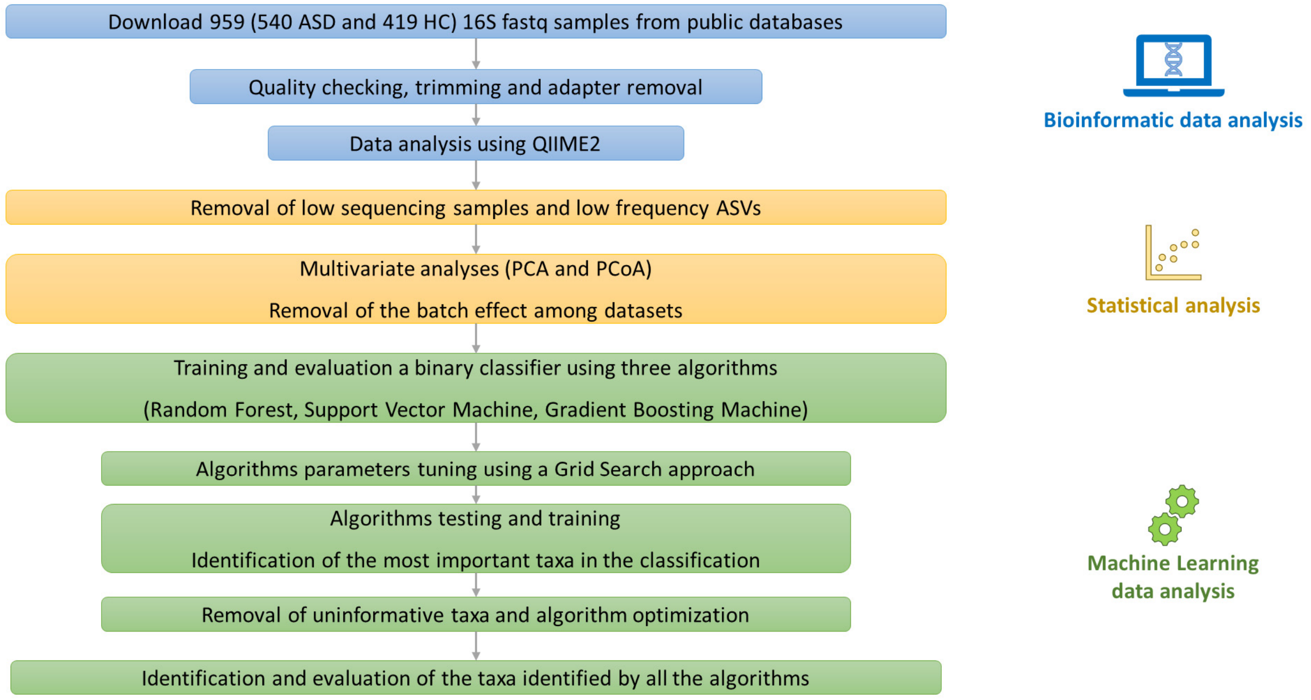

Supplementary Figure S1. Flowchartof the analysis of the implemented strategy.

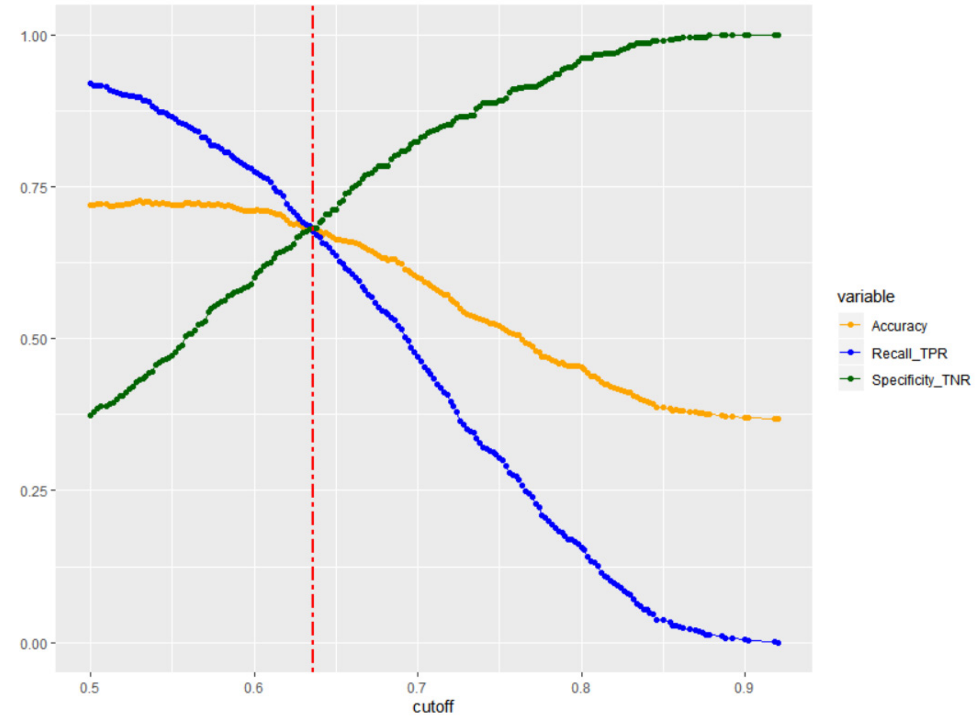

Supplementary Figure S2. Graphical representation of the procedure used to select the best probability threshold (cutoff) for the classifiers. When an algorithm is evaluated, a sample is classified as a “Positive” sample if the probability of being classified as “Positive” is equal to or greater than 50%. Otherwise, the sample is classified as a “Negative” sample. In this study, the “Positive” and “Negative” samples are represented by ASD and HC samples, respectively. The value of this cutoff can lead to different True Positive and True Negative values. Increasing the cutoff, the TPR values (blue line in the graph) will increase, while the TNR (green line) will decrease. The red dotted line represents the best possible cutoff, in which TPR and TNR show the same value. We considered this value asthe best compromise to create a classifierthat recognizes ASD and HC samples. The yellow line represents the accuracy of the algorithm.

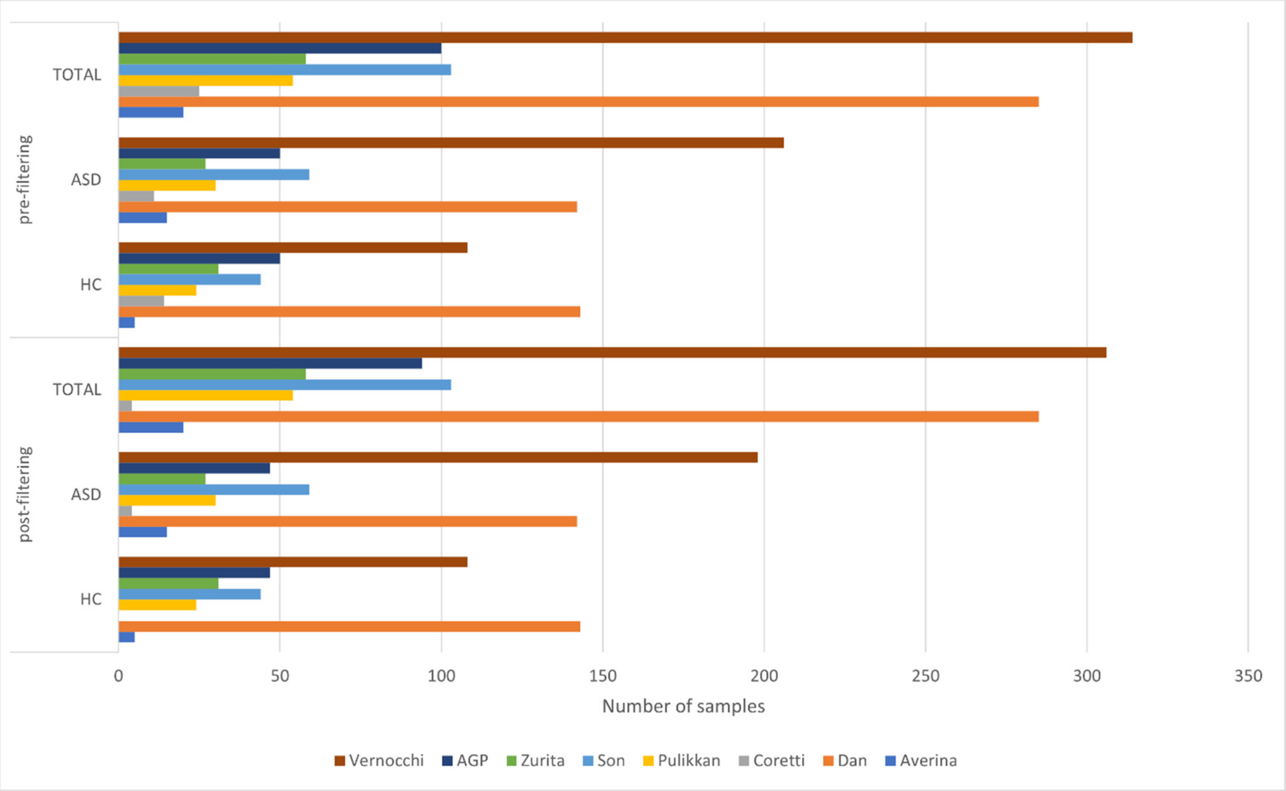

Supplementary Figure S3. Number of samples for each dataset pre and post filtering procedure.

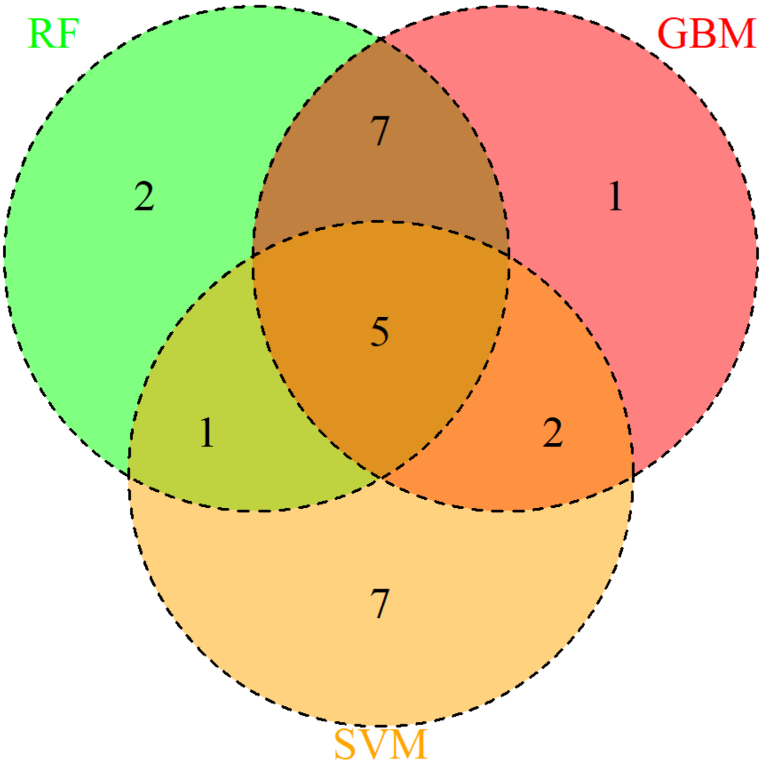

Supplementary Figure S4. Venn Diagram of the feature identified by each algorithm
